# Drug reaction with eosinophilia and systemic symptoms syndrome in a patient taking phenytoin and levetiracetam: a case report

**DOI:** 10.1186/1752-1947-7-2

**Published:** 2013-01-03

**Authors:** David Jeffrey Hall, Jason Steven Fromm

**Affiliations:** 1 College of Medicine, University of Florida College of Medicine, 1600 SW Archer Road, PO Box 100277, Gainesville, FL, 32610-0277, USA; 2 Department of Medicine, University of Florida College of Medicine, 1600 SW Archer Road, PO Box 100277, Gainesville, FL, 32610-0277, USA

**Keywords:** Allergy, Anti-epileptic, DRESS syndrome, Hepatitis, Hypersensitivity reaction, Levetiracetam, Phenytoin, Rash

## Abstract

**Introduction:**

Drug reaction with eosinophilia and systemic symptoms syndrome is a potentially life-threatening hypersensitivity reaction with rash, fever, and internal organ involvement, often hepatitis, occurring most commonly two to eight weeks after initiation of a medication. The present case is an example of severe and potentially life-threatening hepatitis as a manifestation of drug reaction with eosinophilia and systemic symptoms syndrome.

**Case presentation:**

We report a case of anti-epileptic-induced drug reaction with eosinophilia and systemic symptoms syndrome in an 18-year-old African-American man who presented with a five-day history of rash, periorbital and upper extremity edema, hepatitis and fever. Laboratory findings revealed an atypical lymphocytosis, eosinophilia, and elevated serum transaminases. No drug allergies were reported at the time of presentation, but phenytoin and levetiracetam therapy had been initiated five weeks prior to hospital admission for new-onset seizures. Both medications were discontinued on hospital admission, and after three days of high-dose corticosteroid therapy the patient experienced resolution of both his symptoms and laboratory markers of inflammation.

**Conclusion:**

Given the significant mortality attributed to drug reaction with eosinophilia and systemic symptoms syndrome, medical personnel should be aware of the potential for this severe hypersensitivity reaction and should ensure close follow-up and offer anticipatory guidance when beginning any new medication, particularly anti-epileptic therapy. Early recognition of drug reaction with eosinophilia and systemic symptoms syndrome and initiation of appropriate therapy are imperative in limiting morbidity.

## Introduction

Drug reaction with eosinophilia and systemic symptoms (DRESS) syndrome, also known as drug-induced hypersensitivity syndrome (DIHS), is an under-recognized and potentially life-threatening hypersensitivity reaction associated with a variety of medications, many being anti-epileptics. Patients with DRESS syndrome typically present with rash, swelling, fever, and systemic manifestations such as a severe transaminitis [1]. In most cases, a patient’s face, trunk, and upper extremities are affected by a rash which is at first morbilliform then gradually transitions to maculopapular, and finally can progress to edema of the face, particularly in the periorbital region. Although rash and eosinophilia are commonly seen in hypersensitivity reactions, the defining characteristic of DRESS syndrome is organ dysfunction, most commonly of the liver, kidneys, heart, or lungs. These patients are typically found to have started one of a few select medications in the past two to eight weeks (Table 1) with aromatic anti-epileptics being the most commonly implicated [2-6]. Non-aromatic anti-epileptic medications such as a topiramate, ethosuximide, and levetiracetam were traditionally thought to be safer; however, a recent case report described DRESS syndrome in a patient taking only levetiracetam [7]. Although the true incidence is unknown, DRESS syndrome has been estimated to occur in approximately one out of 1000 to 10,000 new users of anti-epileptic medications and is more commonly reported in African-American men [1,8,9]. Vitamin D deficiency has been implicated as a possible contributor to the pathogenesis of DRESS due to its protective effects against inflammatory and auto-immune conditions, and because vitamin D deficiency occurs more frequently in people with darker skin phenotypes [10].

**Table 1:** Drug groups commonly associated with drug reaction with eosinophilia and systemic symptoms syndrome

**Drug Groups:**	**Specific Examples:**
**Anticonvulsants**	**phenytoin, carbamazepine, phenobarbital, lamotrigine, valproate**
**Antidepressants**	**despiramine, amitriptyline, fluoxetine**
**Sulfonamides/sulfones**	**dapsone, sulfasalazine, trimethoprim-sulfamethoxazole**
**Anti-inflammatories**	**piroxicam, naproxen, diclofenac, sulindac, ibuprofen**
**Anti-infectives**	**abacavir, nevirapine, linezolid, doxycycline, nitrofurantoin**
**Angiotensin-converting enzyme inhibitors**	**captopril, enalapril**
**Beta-blockers**	**atenolol, celiprolol**

DIHS was originally described in 1950 by Chaiken *et al*. as a triad of fever, rash, and multi-organ failure [11]. The acronym DRESS was then put forth by Bocquet *et al.* and often includes hepatitis, pericarditis, interstitial nephritis, or interstitial pneumonitis [1,12]. Isolated elevation of liver transaminases is the most common laboratory manifestation of hepatitis in DRESS syndrome. In severe cases it can progress to fulminant liver failure, occurring in as many as 10% of cases and accounting for the principle cause of mortality in patients affected by DRESS syndrome [1]. Although the pathophysiology of DRESS syndrome remains unknown, eosinophilic infiltration is probably the mechanism for involvement of organs such as the liver and kidneys [2].

Prompt recognition and removal of the offending agent is the key to limiting further hepatic damage, although hepatitis may significantly worsen even after discontinuation of the drug and may take months to resolve completely. Although no randomized-controlled therapy trials have been done, corticosteroids are utilized in many reported cases [3-5,8]. No specific therapeutic regimen or dosing has been shown to be more beneficial than another, but it is important that therapy is continued for long enough in order to prevent the possibility of relapse. The following case report demonstrates the necessity of prompt recognition and initiation of appropriate therapy in preventing the potential sequelae of DRESS syndrome.

## Case presentation

An 18-year-old African-American man presented with a five-day history of pruritic, maculopapular rash with associated periorbital swelling, fever, and transaminitis. Five days prior to presentation he noted pruritis and rash over his extremities, which over the next several days progressed to his chest, back, and face. He had a history of seizures that began 35 days prior to this admission treated with phenytoin extended-release ER 100mg daily and levetiracetam 500mg twice a day. After investigation, no specific focus or etiology of his seizures had been identified. He has had decreased verbal and reading skills since early childhood, but details about his delivery and early development are unclear because he was adopted. The patient had no other significant past medical history, drug allergies, or alcohol use. Review of systems was positive for non-productive cough, fever, and tea-colored urine, and negative for chest pain, abdominal pain, shortness of breath, nausea, vomiting, weight-loss, chills, or any recent altered mental status.

On examination, the patient was febrile to 40.2°C (104.4°F) with a heart rate of 88 beats/minute, respiratory rate of 18, and blood pressure of 110/55mmHg. The patient was well nourished, well developed, alert and well oriented, and appeared uncomfortable but not in distress. A fine exanthematous rash was noted on the face, upper, and lower extremities in sun-exposed areas without involvement of the oral mucosa, palms, or soles. There was profound periorbital edema that prevented eye-opening. His abdomen was soft and non-distended with no tenderness, guarding, or hepatosplenomegaly. No focal deficits were appreciated on neurological examination. At this point the differential diagnosis included drug-induced hypersensitivity, erythema multiforme, toxic epidermal necrolysis, vasculitis, an exanthem due to viral infection such as Epstein–Barr virus (EBV), cytomegalovirus (CMV), and human immunodeficiency virus (HIV), and auto-immune conditions such as systemic lupus erythematosus.

Laboratory results revealed a white blood cell count of 7.9 thousand/mm^3^ (normal from 4.0 to 10.0 thousand/mm^3^), with 60% neutrophils, 8.0% lymphocytes, and 4.0% eosinophils (absolute 0.32 thousand/mm [3]). His free phenytoin level on admission was 0.4mcg/mL (therapeutic from 1.0 to 2.0mcg/mL). His basic metabolic panel was within normal limits. Hepatic function panel revealed an aspartate aminotransferase of 778U/L (normal from 0 to 37), and alanine aminotransferase (ALT) of 1274U/L (normal from 0 to 41). Acetaminophen and salicylate levels were below detectable limits. Evaluation for acute and chronic hepatitis with serologies was negative for hepatitis A, B, and C. An extensive workup was performed including electrocardiogram and echocardiogram which were negative for abnormalities. EBV, CMV, and HIV testing were all negative, as were the results of tests for antinuclear antibodies (ANA). The patient was admitted to our hospital with a presumptive diagnosis of drug-induced hypersensitivity. All medications were discontinued and the patient was monitored for signs of clinical recovery.

**Figure 1:**
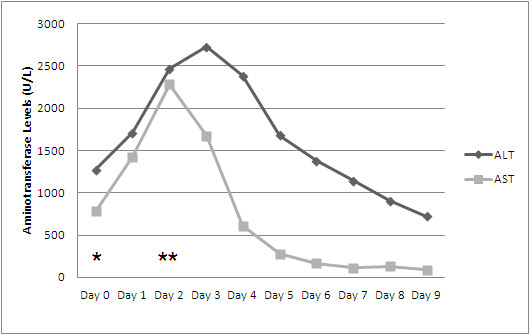
**Serum measurements of alanine aminotransferase (ALT) and aspartate aminotransferase (AST) during the patient’s admission.** Normal reference ranges are from 0 to 41U/L for ALT and from 0 to 37U/L for AST. Day 0 (*) represents the day of admission and discontinuation of phenytoin. Corticosteroid therapy was begun on Day 2 (**).

On hospital day 1, the patient’s condition worsened with increased facial swelling and rash extending to his chest and abdomen. He began to show signs of liver synthetic dysfunction with an elevated prothombin time and international normalized ratio as well as an increasing transaminitis. A repeat complete blood count showed an atypical lymphocytosis and eosinophilia at 8.0%. Because of his deteriorating condition, the patient was started on dexamethasone 4mg orally four times daily. On hospital day 2, the patient showed a marked clinical recovery. Despite improvement in the patient’s rash, his levels of transaminases continued to climb, necessitating hepatology consultation to assist with evaluation for transplantation. On hospital day 3 his levels of transaminases began to improve, and by day 8 his transaminitis had substantially resolved (Figure 1) and he was discharged home on prednisone 50mg to be taken once a day until follow-up with a hepatologist.

Follow-up five months after discharge revealed that the patient was doing well with no recurrence of his rash or other symptoms, no seizures, and normalization of his serum transaminases. He experienced no flare after corticosteroid tapering or withdrawal and to date has not had any hepatic sequelae.

## Discussion

DRESS syndrome is an often under-diagnosed and under-recognized severe type IV (delayed type) hypersensitivity reaction that can occur with any medication but most commonly in response to aromatic anticonvulsants [1,2,6,9,10,12,13]. Like most severe allergic reactions, DRESS syndrome involves rash, diffuse swelling, as well as eosinophilia [1,2,11,14]. The hallmark of DRESS syndrome, however, is the presence of systemic manifestations such as inflammation of the liver, kidneys, heart, or other organs [1,12,13]. Although no formal diagnostic criteria have been widely accepted, a Japanese working group in 2007 established a set of diagnostic guidelines requiring the following: first, maculopapular rash developing greater than three weeks after starting a drug; second, prolonged clinical symptoms two weeks after discontinuation of the causative drug; third, fever greater than 38°C; fourth, liver abnormalities (including ALT greater than 100U/L); fifth, leukocyte abnormalities (either leukocytosis greater than 11×10^9^/L, an atypical lymphocytosis, or eosinophilia greater than 1.5×10^9^/L); sixth, lymphadenopathy; and seventh, human herpesvirus 6 (HHV-6) reactivation [2]. The patient described here met all of these described criteria for a diagnosis of DRESS. Although he had no palpable lymphadenopathy, an abdominal computed tomography scan confirmed profound retroperitoneal lymph node enlargement. Finally, a qualitative deoxyribonucleic acid (DNA) assay revealed the presence of HHV-6 type B in the patient’s blood, indicating the reactivation of HHV-6 associated with the patient’s DRESS syndrome [2,15,16].

Alternatively, Kardaun *et al.* of the Severe Cutaneous Adverse Reactions (RegiSCAR) study group published a scoring system in 2007 which has also been widely used to evaluate potential cases of DRESS syndrome [14]. The criteria for this system include: first, fever greater than 38.5°C; second, enlarged lymph nodes; third, eosinophilia; fourth, atypical lymphocytosis; fifth, skin involvement; sixth, organ involvement; seventh, resolution greater than 15 days; and eighth, evaluation of other causes (ANA, blood cultures, serology for hepatitis A virus, hepatitis B virus, hepatitis C virus, and chlamydia and/or mycoplasma). Using this scoring system, a final score of less than two indicates no case, a final score of between two and three indicates a possible case, a final score of between four and five indicates a probable case, and a final score of greater than five indicates a definite case. The patient in this case report had a score of six points (one each for lymphadenopathy, eosinophilia, atypical lymphocytosis, skin rash suggestive of DRESS, liver involvement, and evaluation of other potential causes), indicating a ‘definite case’ of DRESS per the RegiSCAR scoring guidelines.

An important question to consider is which medication was actually the source of the patient’s reaction, as he had been started on phenytoin and levetiracetam within days of each other due to recurring seizures on phenytoin alone. Although DRESS was originally described in response to phenytoin and it has been one of the most common causative medications, Gómez-Zorrilla *et al.* published a case report earlier this year (2012) of a patient presenting with DRESS syndrome who took no medications other than levetiracetam [6,7,11]. If the patient were to again require anticonvulsant therapy, it would be prudent to avoid use of both phenytoin and levetiracetam, and to opt instead for an alternative non-aromatic anticonvulsant.

Prompt recognition of the adverse drug reaction and discontinuation of offending medication are imperative steps in limiting the progression of DRESS syndrome. Pharmacological treatment of DRESS syndrome has to date not been studied with randomized controlled trials and instead has been established on the basis of case reports and retrospective analysis. Systemic corticosteroids have become a mainstay of therapy in severe cases and often produce marked improvement in clinical symptoms and laboratory measures in just a few days after the initiation of treatment [3-5,8]. If symptoms continue to progress despite the use of corticosteroids, other options include intravenous immunoglobulin (IVIG) and/or plasmapheresis [6].

The French Society of Dermatology published a report in 2010 outlining a consensus on therapeutic management of DRESS [17]. They recommend the use of systemic corticosteroids at a dose equivalent to one mg/kg/day of prednisone in patients with any sign of severity including: transaminases greater than five times normal, renal involvement, pneumonia, hemophagocytosis, or cardiac involvement. They further recommend the use of IVIG at a dose of two g/kg over five days for a patient with life-threatening signs such as renal failure or respiratory failure. In addition, they propose the use of steroids in combination with ganciclovir in patients with signs of severity and confirmation of a major viral reactivation of HHV-6. However, because anti-HHV-6 immunoglobulin G titers are not currently widely available in all hospitals and laboratories, results often take several days or weeks to confirm viral reactivation. Because time is an important factor in the treatment of DRESS, it is not recommended to delay definitive therapy in order to confirm a major viral reactivation. Further study and randomized controlled trials of these and other potential pharmacologic therapies will be important in establishing a standard of care and to improve understanding of how best to treat patients affected by DRESS syndrome.

## Conclusion

Given the significant mortality attributed to DRESS syndrome, clinicians should be aware of the potential for this severe hypersensitivity reaction particularly in starting any new anti-epileptic medication. In patients presenting with skin rash and systemic abnormalities after a recent change in medications, physicians should consider DRESS syndrome as a possible diagnosis and switch to more aggressive therapy if removal of the offending agent does not result in clinical improvement. Further study of potential pharmacological therapies is warranted given the significant morbidity associated with DRESS syndrome.

## Consent

Written informed consent was obtained from the patient and his legal guardian for publication of this case report and any accompanying images. A copy of the written consent is available for review by the Editor-in-Chief of this journal.

## Competing interests

The authors declare that they have no competing interests.

## Authors’ contributions

DH analyzed and interpreted the data regarding the patient’s condition and wrote the first draft of the manuscript. JF examined and administered treatment to the patient and was a major contributor in writing and editing the manuscript. Both authors read and approved the final manuscript.
